# Looking through different lenses at meeting places in disadvantaged Dutch neighbourhoods: A qualitative participatory study assessing needs, barriers and success elements for women with non-Western migration backgrounds and teenage girls

**DOI:** 10.1371/journal.pone.0356072

**Published:** 2026-09-09

**Authors:** Geertje van Wijk, Louise T. C. Mulder, Suzanne van der Geest, Annemarie Kapteijns, Marieke C. E. Battjes-Fries

**Affiliations:** 1 Department of Health, Louis Bolk Instituut, Bunnik, Utrecht, The Netherlands; 2 Department of Research, GGDrU, Zeist, Utrecht, The Netherlands; University of Amsterdam, NETHERLANDS, KINGDOM OF THE

## Abstract

**Objective:**

The living environment can significantly influence health negatively, particularly in disadvantaged neighbourhoods where risk factors and limited resources converge. Social cohesion and inclusive meeting places promote well-being, yet little is known about what makes these spaces accessible and meaningful for specific groups. This study explored how neighbourhood meeting places can better align with the needs of two often underrepresented groups: women with a non-Western migration background and teenage girls (12–18 years old), in four disadvantaged Dutch neighbourhoods.

**Methods:**

A qualitative participatory study was conducted in four municipalities in the Netherlands in March 2024 – June 2025. Data were collected through a neighbourhood orientation, focus groups, PhotoVoice, interviews, and validation sessions with neighbourhood workers, women, and girls, to assess the needs, barriers and success elements regarding meeting places. Thematic analysis was guided by the Movement Friendly Environment model, organizing data into four domains: physical design (hardware), social use (software), organizational structures (orgware), and perception (mindware).

**Results:**

In total, 97 participants contributed: 29 neighbourhood workers, 44 women, and 24 girls. The results, structured according to the four ‘wares,’ revealed a broad range of needs and experiences regarding meeting places. Women and girls valued clean, nearby, and welcoming public spaces with sufficient seating, safety, greenery and gender-sensitive design. Preferred activities included cooking, language learning, informal gatherings, and sports, ideally in familiar, culturally sensitive, and child-friendly settings. Ownership, trust and feelings of safety were important factors in fostering engagement. Barriers included unclear communication, language difficulties, lack of childcare, and feelings of unsafety. Neighbourhood workers played an important role in outreach, facilitation, and empowerment.

**Conclusion:**

Accessible, inclusive and meaningful meeting places for women and teenage girls emerge when physical, social, and organizational elements are aligned and mutually reinforcing, and are integrated with citizen’s perception, participation, engagement, and strong social networks.

## Introduction

The living environment – comprising the spaces in which people live, work, travel, and engage in leisure – influences health significantly [1–3]. A healthy living environment promotes a healthy lifestyle through opportunities for physical activity, offers space for social interaction, and can help reduce the risk of chronic diseases [1,3–5]. Increasing evidence suggests that healthy living environments influence health not only through physical environmental characteristics, such as access to green space, but also through social mechanisms, including social cohesion, feelings of safety, and opportunities for encounter and participation [3,6–8]. Consequently, healthy living environments are increasingly understood as places that support healthy behaviours while also fostering social connectedness and participation [3,6,9]. Furthermore, in light of rising rates of people with a chronic disease, substantial socioeconomic health disparities, and an estimated minimum of 6% of the disease burden attributable to an unhealthy environment, the promotion of healthy living environments has become increasingly urgent [6,7]. It is therefore essential to foster environments that support and promote health.

The extent to which a living environment supports health is partly determined by the neighbourhood in which one resides. There are substantial differences between neighbourhoods in how the environment promotes or hinders health [8,9]. These differences pertain to factors such as air quality, safety, noise pollution, poorly maintained homes, social cohesion and access to green spaces [9–11]. Health-compromising factors are more prevalent in disadvantaged neighbourhoods, where residents often face a cumulative burden of problems that negatively impact their health [9,10,12,13]. Such areas are frequently characterized by higher numbers of residents who are unemployed, have low incomes or debts and rely on social benefits, and with high rates of low literacy and loneliness [9,14–16]. This accumulation of financial and social problems, combined with living in an environment that does not support health, makes it particularly challenging to make healthy choices regarding physical activity, healthy eating, and social interaction.

Social interactions, connections and encounters lie at the core of social cohesion, which is a crucial element for healthy residents and liveable neighbourhoods, especially in disadvantaged neighbourhoods. Strong social cohesion fosters mutual support and a sense of belonging. Social interaction with neighbours can help reduce mental health issues, while increased use of public space contributes to greater safety and healthier lifestyles [7,17,18]. Social cohesion also enhances feelings of purpose and participation in society. Public spaces and neighbourhood meeting places are increasingly recognised as settings through which social cohesion may develop. Francis et al. [19] demonstrated that public spaces contribute to a sense of community by facilitating repeated encounters and shared experiences, while Qi et al. [20] found that public spaces can strengthen social cohesion by promoting social interaction, trust, reciprocity, and belonging. Meeting places in the neighbourhood, such as community centres, sports clubs, playgrounds, or picnic benches, where residents can participate and engage with one another, therefore contribute to strengthening social cohesion and fostering of a health-supportive physical environment [1,7,18,19].

Designing neighbourhood meeting places that are actively used by residents requires a tailored approach, aligned with the needs and characteristics of the specific target group [19–24]. Research increasingly acknowledges that experiences of public and community spaces differ across social groups and that gender-sensitive approaches are needed in the design of neighbourhood environments [24]. These experiences can differ because of several factors, such as safety and privacy concerns, gendered spatial design (often favouring boys), and limited involvement of target groups in the development of such spaces [23–26]. However, despite growing attention to gender and inclusion in the design of public spaces, relatively little research has examined what makes neighbourhood meeting places meaningful and accessible for specifically women with a non-Western migration background and teenage girls [24–28]. As a result, limited evidence is available to guide municipalities and community organisations in developing meeting places that are responsive to the needs of these groups, potentially limiting their participation in and use of such places [24–26]. Insight into the specific needs and preferences of women with a non-Western migration background and teenage girls regarding neighbourhood meeting places is therefore valuable, particularly in disadvantaged neighbourhoods where opportunities for social participation and social cohesion may be under pressure. The achieved aim of this study was therefore to explore which elements make neighbourhood meeting places work for women with a non-Western migration background and teenage girls (ages 12–18) in disadvantaged neighbourhoods.

## Theoretical framework

Research on public space has increasingly moved beyond viewing places merely as physical environments. Carmona [29,30] argues that the functioning of public spaces cannot be understood solely through their design but instead emerges through the continuous interaction between spatial characteristics, social practices, and modes of management and governance. This multidimensional perspective aligns with the distinction between hardware, software, and orgware, which originated from systems approaches to technological and organisational development [31], and was later adopted in urban planning and placemaking contexts [32]. Hardware refers to the tangible and spatial characteristics of places, software concerns the activities and social interactions occurring within them, and orgware relates to the organisational arrangements of the places. Within placemaking discourse, scholars and practitioners increasingly emphasise that meaningful places emerge through the interaction of these dimensions rather than through physical design alone [32].

Practice-oriented placemaking organisations have similarly embraced this integrated perspective. For example, STIPO, an international urban development and placemaking organisation, advocates combining interventions aimed at the physical environment, social activation, and local organisational capacity to strengthen public life and neighbourhood vitality [33]. Such approaches illustrate how the interaction between hardware, software, and orgware has become an important guiding principle in the design and management of inclusive public spaces.

Building upon these developments, the Dutch Knowledge Centre for Sport and Physical Activity translated this way of thinking into the ‘Beweegvriendelijke Omgeving’ (BVO) model [34]. Although originally developed to understand and stimulate physical activity within neighbourhood environments, the BVO model provides a comprehensive framework for analysing how places function more broadly. The model distinguishes the same three interconnected dimensions. Within this model, hardware refers to the physical aspects and availability of facilities where people can meet and engage in activities. This includes both indoor and outdoor spaces and encompasses aspects such as accessibility, proximity, aesthetics, and the presence of appropriate amenities. Software concerns what takes place within these physical settings and includes the interactions between residents, the activities offered, communication surrounding these activities, and the guidance or facilitation provided. Orgware relates to the organisational structures and coordination required to facilitate and sustain participation. It encompasses governance arrangements, the involvement of relevant organisations, the mobilisation of resources, engagement with residents, and the roles residents themselves fulfil within these contexts [34].

While the BVO model has primarily been applied within the context of physical activity and sport participation, the underlying framework is useful for understanding neighbourhood meeting places. Similar to physical activity settings, meeting places might also rely on an interplay between suitable environments, meaningful activities, and sustainable organisational support. Applying the BVO model to neighbourhood meeting places therefore allows for a systematic examination of the multiple factors that may facilitate or hinder participation.

In addition, the debates described by Carmona [29,30], also suggest that physical, social, and organisational dimensions alone may not fully explain why people choose to use, avoid, or identify with particular places. Discussions about inclusion, exclusion, safety, atmosphere and control ultimately concern how individuals perceive and experience public spaces. Building on this notion, placemaking scholars have proposed a fourth dimension: mindware [32]. Mindware refers to the meanings, perceptions, and emotional responses attached to places and concerns how people perceive and imagine their environment. It encompasses aspects such as values, trust, identity, feelings of safety, belonging, and whether people experience a place as welcoming and intended for them [32].

Although mindware has increasingly been acknowledged within placemaking discourse and practice, empirical studies explicitly incorporating this fourth dimension remain limited. Previous studies have shown that perceptions of social acceptance, emotional safety, recognition, and belonging strongly influence whether people engage in community activities and utilise local facilities [35–38]. Focusing exclusively on physical, social, and organisational conditions may therefore overlook important experiential mechanisms underlying participation. Hence, mindware is also taken into account in this study.

## Materials and methods

### Research setting

This study was conducted between March 2024 and June 2025 in four disadvantaged neighbourhoods in the municipalities of Utrecht, Amersfoort, Nieuwegein, and Zeist, all located in the Dutch province of Utrecht. Although no uniform definition exists, disadvantaged neighbourhoods in the Netherlands are typically characterized by infrastructural, economic, and social challenges, such as school dropout, youth unemployment, crime, inadequate integration of newcomers, and limited emancipation opportunities for non-Western women [39,40]. These areas often receive additional governmental support. The four municipalities all participated in the ‘Regio Deal Vitale Wijken’ (Vital Neighbourhoods Regional Deal), a provincial initiative aimed at strengthening vulnerable neighbourhoods, with a focus on meeting places as a key element of neighbourhood vitality [41]. The municipalities were invited to take part in this study through their involvement in this programme and voluntarily agreed to participate. Subsequently, each municipality identified a neighbourhood in which they considered additional attention and investment in neighbourhood meeting places to be particularly relevant, to include in this study.

The Medical Ethics Review Committee of Amsterdam University Medical Centres declared the current study in writing as ‘non-WMO’, indicating that the Medical Research Involving Human Subjects Act (WMO) does not apply to this study (number: 2024.0557).

### Participants

Women with a non-Western migration background, teenage girls and professional neighbourhood workers were eligible to participate in this study. Inclusion criteria for women with a non-Western migration background included being a resident of one the four participating neighbourhoods, aged >18 years and preferably first-generation migrants. In case women were from second-generation, they were also included. Participants with limited or no proficiency in Dutch were included as well. No exclusion criteria were set for income, length of residence, or other characteristics. A non-Western migration background in this study was defined as a person originating from a country in Africa, South America or Asia (excl. Indonesia and Japan) or from Turkey as described by the Central Statistical Office of the Netherlands [42].

Inclusion criteria for teenage girls were being aged 12–18 years and being a resident in the one of the four participating neighbourhoods. In the neighbourhood of the municipality of Utrecht, teenage girls were excluded from the study since neighbourhood- and youth workers working with teenage girls had indicated beforehand that they were unable to participate due to lack of time, making recruitment and data collection not feasible. In the three other neighbourhoods all three groups participated.

Neighbourhood workers were included who were employed within the physical or social domain, the municipality, welfare organisations or schools in the participating neighbourhoods, and worked or were in contact with the women with non-Western migration background and/or teenage girls.

There was no pre-established relationship between the researchers and the participants. Also, the participants did not know of any personal goals or information about the researchers, nor were any characteristics of the researchers reported.

### Recruitment

The recruitment found place between 01/05/2024 and 28/02/2025. Eligible neighbourhood workers were selected through a tour in the participating neighbourhood (see next section), suggestions for contacts made by the municipalities, desk research focused on the available organizations active in the neighbourhood, and a snowball sampling technique. Neighbourhood workers were contacted through phone calls and emails and asked whether they were 1) interested in participating in the study themselves and 2) willing to facilitate recruitment of women and girls.

Recruitment of women and girls was facilitated through the existing networks of neighbourhood workers and youth workers, who were in contact with the target groups. Recruitment of women and girls was performed differently in each of the four neighbourhoods and was strongly dependent on the size and availability of the existing network of neighbourhood workers.

To recruit the women and girls, flyers were developed containing information regarding the goal of the study and practical information about participating. The flyers were specifically targeted to each group by adjusting lay-out and language. For the women, flyers were available in Dutch, English and Arabic, for girls in Dutch. Flyers were spread by neighbourhood- and youth workers throughout the neighbourhood in community centres, local places such as the library or secondary schools, and online via social media and community web pages. In one neighbourhood the flyers were also spread door to door. In addition, researchers attended a few local activities in schools and community centres themselves to actively recruit women and girls. Lastly, a snowball sampling technique was used to assist in the recruitment of other participants, by letting the women and girls sign others from their network in for the study or take them with to the sessions last-minute.

Women and girls could register for the study via a neighbourhood worker or by filling in a registration form accessed through a QR code on the flyers. Participants provided their name, age, contact details, and neighbourhood of residence. For women, the form also included background questions regarding parenthood and Dutch language proficiency. In practice, the use of the registration form was not always feasible due to limited language or digital skills among some participants. Therefore, in several cases, neighbourhood workers helped participants to fill in the form or informed the researchers directly about participation. Researchers subsequently contacted all participants personally via telephone or email to schedule a focus group or interview.

### Study design

This study employed a participatory action research approach, emphasizing methods that aligned closely with participants’ experiences and actively involved them throughout the research process. Three qualitative methods were used: PhotoVoice, focus group discussions, and interviews. Involving both women and teenage girls in the validation of results further increased compatibility between the research process and participants’ perspectives.

**Fig 1:**
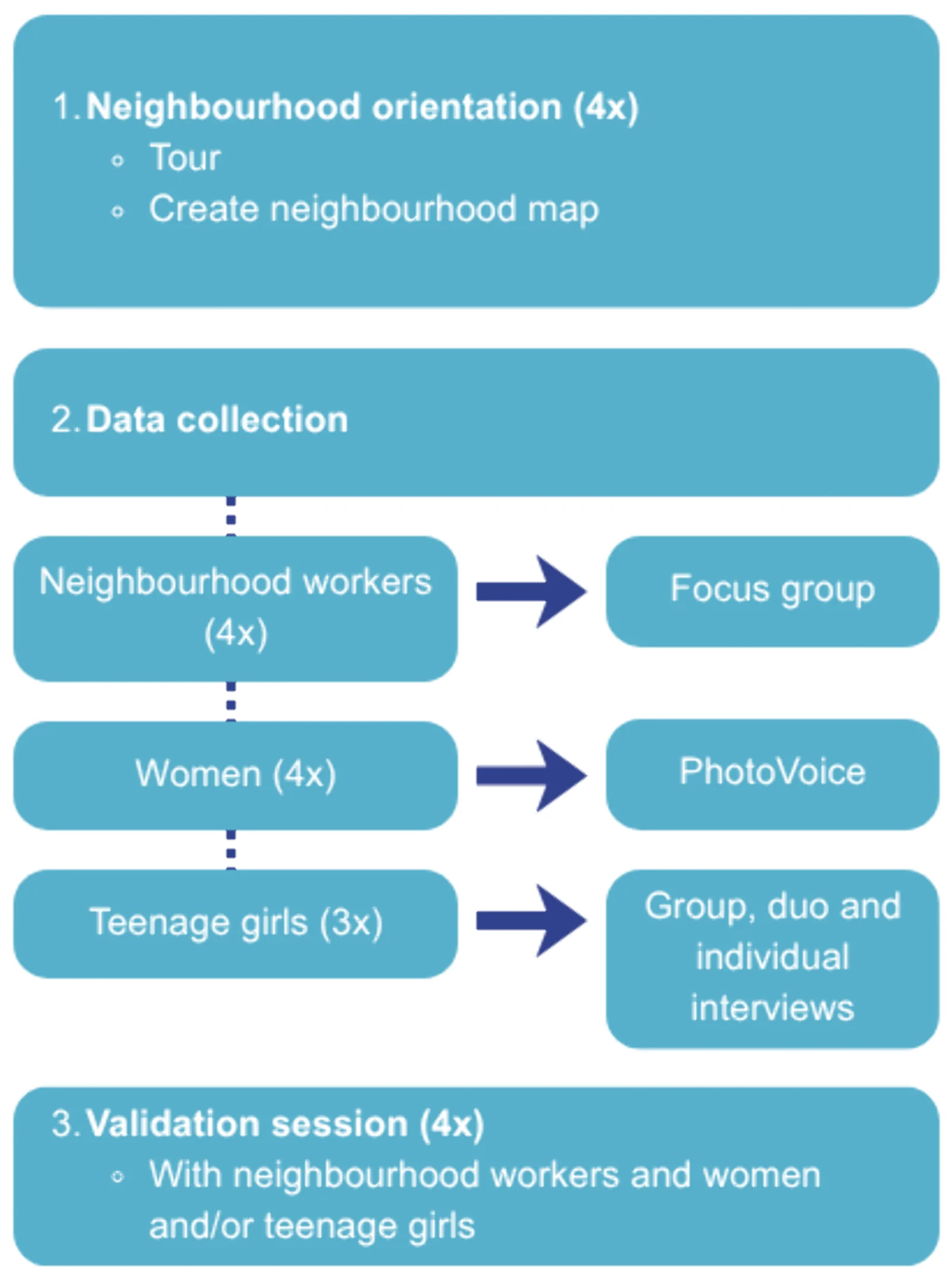
Data collection step by step. The numbers in parentheses refer to the number of municipalities where this took place.

Data was collected in each participating neighbourhood following the steps shown in Fig 1. The research process began with an orientation phase, followed by data collection through PhotoVoice, focus groups and interviews, and concluded with validation sessions to discuss and refine the findings. All steps were conducted by authors GvW, LM and SvdG. With the women and girls, different data collection formats were applied depending on feasibility and participant preferences. Focus groups were preferred over individual interviews for women, as they facilitate collective reflection and discussion of shared experiences [43], while also responding to concerns raised by neighbourhood workers that some women might feel uncomfortable participating in one-on-one interviews. The potential influence of focus group dynamics on participants’ willingness to express differing perspectives was considered. For teenage girls, organizing the focus groups was proved to be more challenging due to limited interest, time constraints related to school, side jobs, difficulties in reaching girls without available youth workers, and the requirement for signed parental consent for participants aged 12–16 that formed an extra barrier for the girls. Therefore, it was decided to conduct interviews with the girls instead of focus groups. Interviews may provide more space for personal experiences but also involve different participant-researcher dynamics. The implications of using multiple methods are further reflected upon in the Strengths and limitations section.

### Data collection and procedure

#### Neighbourhood orientation.

In May-June 2024, each participating neighbourhood was explored through site visits and guided tours led by one or more neighbourhood workers and/or female residents. Based on their input and the visited locations, a local map was created to visualize key meeting places. These maps were later used in focus groups or interviews with the neighbourhood workers, women, and teenage girls. Each meeting place was numbered and supplemented with photos.

#### Focus groups, PhotoVoice and (group) interviews.

*Neighbourhood workers.* In Juli-August 2024, focus groups with neighbourhood workers were held in local venues such as community centres or town halls. Their goal was to explore how women with a non-Western migration background and teenage girls meet and interact within the neighbourhood, from the perspective of the neighbourhood workers. Prior to participation, the neighbourhood workers received information about the study through an online meeting, one per municipality. The focus groups lasted 1.5 to 2 hours, were audio-recorded, and written informed consent was obtained from all participants at the beginning of the session.

A semi-structured topic guide was used. Discussions began by exploring whether the workers recognized the described neighbourhood characteristics and whether these matched their own observations. Then, they shared their perspectives on the experiences of women and teenage girls in the neighbourhood. The local maps created during the orientation phase were used to prompt discussion on the presence and use of specific meeting places. Participants reflected on pleasant and unpleasant locations, behavioural dynamics, and the needs and wishes of the target groups regarding existing and future meeting places.

*Women with a non-Western migration background.* In September-October 2024, after the focus groups with neighbourhood workers, the focus groups with the women were held, using the PhotoVoice method. PhotoVoice is a community-based participatory approach that enables participants to articulate their experiences and environments through photography. It is particularly suitable for groups with limited language proficiency, as it supports visual storytelling and dialogue with policymakers. It offers valuable insights into daily living environments through the participants’ perspectives [44].

The PhotoVoice sessions in this study took place in familiar settings for the women – such as community centres, schools, or churches – and were guided by two to three researchers, alongside a known neighbourhood worker. When needed, translation was provided by researchers or peers to ensure comprehension. The session began with a short introduction followed by a group discussion on living in the neighbourhood. The women were then introduced to the concept of meeting places and were asked to take photographs outside, reflecting on two questions: (1) Where do you go to meet others in the neighbourhood, and why? and (2) Which meeting places do you experience as unpleasant for meeting others, and why? Researchers accompanied them to capture stories and take notes. Photographs were sent by phone to one of the researchers and immediately printed. The printed photos were discussed in the group to explore experiences, needs, and wishes related to meeting places. A PhotoVoice session lasted about two hours, were audio-recorded, and participants received a €20 gift card as compensation. Written informed consent was obtained from all women at the beginning of the session.

*Teenage girls.* In October-November 2024, data was collected of teenage girls. In one neighbourhood, an informal group interview was conducted with multiple girls that could walk in freely. In the other neighbourhoods, a short individual interview or interview in pairs of approximately ten minutes were held with the girls at secondary schools, community centres, or through online video calling or by phone. The interview guide covered questions about pleasant and unpleasant places to meet, motivations for visiting them, and wishes regarding neighbourhood meeting places. Participants received a €15 gift card for group interviews or a chocolate bar for individual or pair interviews. Interviews were not audio-recorded; instead, researchers recorded oral summaries immediately afterwards for analysis. As the interviews were not audio-recorded, were informal of nature and were short in time, asking informed consent was not applicable.

#### Validation sessions.

In order to discuss and confirm the findings, validation sessions were conducted in each participating neighbourhood, in February 2025. In line with the participatory design, these sessions included previous participants: neighbourhood workers, women, and teenage girls; as well as others who had shown interest. Local policymakers from relevant domains were also invited using contact details provided by the participating municipalities. During the two-hour sessions, findings were presented and discussed in terms of (1) recognizability, (2) lessons learned and potential actions, and (3) how these could inform practical approaches and be shared with others. As compensation, women and teenage girls who attended, received gift cards valued at €20 and €15, respectively.

### Data analysis

Software MAX QDA 24 was used to facilitate data management and analysis. All audio recordings were uploaded, transcribed and reviewed for faults by authors GvW, LM and SvdG. If interviews were not audio recorded during the session, a summary on a voice audio was recorded after having the conversation and uploaded in the analysis program. The transcripts of the focus groups with neighbourhood workers were analysed less comprehensive as the data collected with these sessions merely functioned as input for the focus groups and interviews with the women and girls.

Data from the focus groups, PhotoVoice and (group) interviews were divided among the three researchers in such a way that each recording was coded separately by two researchers. Thereafter their work was compared to see if any uncertainties or disagreements existed. Consensus was resolved by discussion, and extra codes were added or merged if necessary. Segments that were relevant to the research question were identified. A combination of deductive and inductive coding was performed. A coding tree was created based upon the three dimensions of the BVO model, extended with the fourth dimension of mindware. These dimensions were used as overarching themes in the data analysis. A final overview of the codes is available in S1 Table.

After coding, thematic analysis was performed on the data per neighbourhood and per group (women or teenage girl) separately and overall, with all neighbourhoods and groups together. Quotes were identified as relevant if they contained information regarding the relationship between the different dimensions (hardware, software, orgware, mindware) and meeting places in the neighbourhood.

## Results

### Demographic characteristics of participants

In total, 97 people participated in this study; 29 neighbourhood workers, 44 women and 24 teenage girls. In Table 1 the exact numbers are shown per participating municipality. In total, 35 neighbourhood workers were invited to attend the focus groups; 29 eventually participated. The neighbourhood workers occupied a range of roles, such as community networker, neighbourhood sports coach, district advisor, participation facilitator, community police officer, youth worker, child welfare practitioner, social worker, project coordinator, neighbourhood coordinator, area/regional manager and neighbourhood volunteer.

**Table 1:** Number of participating neighbourhood workers, women with non-Western migration backgrounds, and teenage girls per neighbourhood.

	Neighbourhood workers	Women	Teenage girls	Total
Amersfoort	7	11	11	**29**
Nieuwegein	5	8	3	**16**
Utrecht	7	20	n. a.	**27**
Zeist	10	5	10	**25**
**Total**	**29**	**44**	**24**	**97**

A total of 28 women registered via the form: 2 in Zeist, 8 in Nieuwegein, and 18 in Utrecht. In Amersfoort, no women registered through the form. Ultimately, 44 women participated in the PhotoVoice sessions. Estimated is that 84% (n = 37) of all participating women were recruited by a neighbourhood worker or joined spontaneously. Even though some women registered through the form, only 16% (n = 7) of them were actually attending the focus groups. Non-attendances were due to work, holidays or personal reasons. Most participants were mothers, with ages ranging from their early twenties to over seventy (exact ages were not collected for all participants). The women represented diverse backgrounds, including Moroccan, Turkish, Surinamese, Antillean, Eritrean, Syrian, Venezuelan, Afghan, Iraqi, and Guinean. In Utrecht, the majority had a Moroccan background, while in the other municipalities backgrounds were more varied. Most participants had limited proficiency of the Dutch language. When needed, neighbourhood workers or fellow participants served as informal interpreters to support mutual understanding.

In total, 10 teenage girls showed interest by filling in the registration form online, of whom 7 teenage girls from Nieuwegein and 3 teenage girls from Amersfoort. In total, 24 teenage girls attended the (group) interviews. Estimated is that 92% (n = 22) of all the participating teenage girls joined through contact of a youth worker/ neighbourhood worker or spontaneously. Notably, only 8% (n = 2) of the teenage girls who signed up via the registration form have actually participated. Teenage girls had an average age of approximately 14 years; and their ages ranged from 13 to 18. In general, all participants were eager to tell and share their experiences around the subject meeting in the neighbourhood.

### Use of meeting places

Women indicated that they spend time meeting others across various locations within the neighbourhood. They expressed to spend their time often at child-friendly places, such as benches near playgrounds or parks, or at schools, urban farms, marketplaces, libraries, and local community centres. Teenage girls indicated that they like to hang out in the neighbourhood on benches in parks, playgrounds, at the football court, shopping centre or at the local community centre. They used these places to chat, relax or to do sports.

### Needs, barriers and success elements

The results are organized into the empirical themes that emerged from the data. Within each theme, the domains of hardware, software, orgware and mindware are described in an integrated manner where relevant, reflecting their interconnected nature. Together, these themes capture the success elements, as well as the needs and barriers related to neighbourhood meeting places for women and teenage girls. The chapter concludes with an overview table that maps the themes onto the four domains. Most findings stem from focus groups and interviews with participants; relevant insights from neighbourhood workers have been incorporated where applicable. Quotes beginning with ‘W’ refer to quotes stated by women, a beginning with ‘TG’ stands for teenage girl.

#### Attractive and accessible spaces.

Both outdoor and indoor environments influenced whether women and teenage girls felt comfortable to spend time at meeting places and interact with others. They expressed that their willingness to visit and engage in public or communal spaces were strongly influenced by how these spaces made them feel. Women and teenage girls across all neighbourhoods highlighted the importance of clean, inviting, and accessible public spaces. These contribute to an increased sense of safety and user-friendliness. Well-maintained green areas, such as parks, were particularly valued for their calming ambiance, recreational opportunities, and cooling effect in summer. Conversely, litter, broken benches, or poorly maintained areas discouraged use by women and girls. Some women described feelings of shame as a result of litter in the neighbourhood that prevented them from inviting others into their neighbourhood.

*“Garbage, in front of your apartment building. Then you come there with your own bag, and you see that. Then you think oh well, I’m part of this too, so I’m complicit.” (W18, municipality 1)*

Beyond physical maintenance, participants emphasized that a space also needed to feel welcoming. The mere availability of a meeting place did not automatically result in its use. Some locations were experienced as uninviting because of their atmosphere, the presence of groups that do not feel relatable, or the character of the building itself. For example, locations dominated by men or older adults may feel less appealing to younger women or teenage girls. Additionally, exclusive use of spaces by certain women’s groups and hosting events in religious venues misaligned with personal beliefs sometimes limited participation.

*“I honestly don’t go there. I sometimes see people sitting there, mostly elderly, having coffee. No, that’s not my crowd.” (W2, municipality 4)*

Indoor places, such as rooms in community centres, should be spacious and suitable for both women and their children. For instance, regarding the design of the physical space, the availability of a kitchen would be appreciated in case of cooking activities or big windows to supervise the children playing outside. Privacy was also important for women, with preferences for enclosed or separable areas (e.g., using curtains), as well as warm, and homely indoor spaces that feel comfortable and safe. Teenage girls valued spaces without teenage boys present, where they felt ownership and a sense of belonging.

*“Something just for girls, without any boys around.” (TG7, municipality 2)*

Teenage girls also noted a lack of designated outdoor spaces, such as chill-out spots or smoking shelters, where they could socialize freely and informally without adult supervision or pressure to conform to others’ expectations.

*“In the past, I always socialized with people. Now, I never talk to anyone anymore. Everyone stays at home. We also don’t really have places. At least, I am not aware that there are meeting places.” (TG3, municipality 2)*

Overall, participants described attractive physical environments as important not only because of their practical qualities, but also because they contributed to feelings of comfort, belonging, safety, and ownership, making meeting places more inviting for social interaction.

#### Amenities located close to home.

Amenities located close to home were highly valued, as women and girls typically carried out daily routines within their neighbourhoods and women tend to move through neighbourhoods by walking. Nearby parks, playgrounds, and community centres also contributed to a lively atmosphere according to the women and girls, encouraging social interaction through the natural movement of people in and out of spaces.

#### Sufficient and strategically located seating places.

Across all neighbourhoods, women and teenage girls emphasized the need for sufficient and strategically placed seating, supporting physical comfort and creating opportunities for spontaneous encounters. The function of seating therewith extended beyond providing places to rest; it facilitated social interaction and encouraged people to remain in public spaces for longer periods. For women, benches near playgrounds enabled informal supervision while offering opportunities for rest and conversation. Shaded seating and picnic tables were particularly appreciated, allowing for social moments with food and drink.

*"When my children are playing, that is the moment for me to talk to other mothers." (W6, municipality 1)*

*"When my children are playing in the park, I’d like to sit nearby. I watch them a bit and rest." (W1, municipality 4)*

For teenage girls it was appreciated to have seating places that provide some privacy from the rest of the surroundings and offer shelter from different weather conditions.

*“We want seating areas specifically intended for us. Additionally, sufficient distance between benches is important, as it provides privacy.” (TG 2, municipality 3)*

#### Safe and diverse play and sports areas.

Both women and girls expressed the need for diverse, safe, and age-appropriate play and sports areas within their neighbourhoods. Sufficient playgrounds for different age groups close to home were desired. Women and girls pointed out that cultural and age-sensitive aspects played a role in whether they felt welcome and whether the setting matched their interests, standards, and values. This influenced their levels of engagement in meeting places positively, both public and indoors. Several women experienced the equipment as unsafe, impractical or not suitable for the intended age group. For instance, the tiles used at the ground at the playground or the closeness of the road was considered as unsafe. In addition, several women and girls valued accessible sports facilities, which made it easier and more affordable for them to be physically active. An example of an accessible sports facility was outdoor fitness equipment that was not physically too heavy. This was in contrast with the neighbourhood workers who thought that the outside fitness equipment was not ideal or safe for the women (regarding privacy).

*“The gym is just unaffordable. So, I said to my friend let’s just go there [outdoor fitness equipment]. It’s fun! It’s a really nice spot. So, I think it’s great that the municipality provides that.” (W1, municipality 3)*

For teenage girls, football cages and modular play containers were frequently mentioned as pleasant meeting places, because they support both social connection and physical activity. However, it was important that the girls felt welcome to join such places, also when the boys are playing. The lack of feeling welcome on certain places such as the football playground or the general negative image of ‘loitering youth’, brought a sense of misplacement. Besides, water taps available at such playing areas were mentioned multiple times as important element, to prolong sport and play time outside.

#### Activities for women.

Women reported enjoying community centres offering activities like cooking, sewing, and creative hobbies. Other appreciated activities for women included sports, gardening, walking, activities around specific themes, seasonal or culturally based (with children). Swimming, particularly women-only sessions, was mentioned frequently, though access was often limited due to waiting lists. Walk-in sessions organised on a regular basis with coffee or tea, especially in familiar locations like schools, were appreciated for their accessibility. Informal conversations about parenting or cultural topics were seen as enjoyable. As for the location for these activities, women emphasized the importance of welcoming, homely indoor spaces that feel warm and safe, and thereby contribute to a sense of comfort and belonging.

*“On Friday morning we have a coffee hour in the church with the women. That is pleasant, chatting nicely and exchanging things, and the children can be there too.” (W11, municipality 2)*

Child-friendly settings were considered essential, either by allowing children to participate or by providing childcare facilities. When childcare was unavailable, women explained that they were often unable to attend activities or were restricted to locations where children were welcome. Scheduling activities immediately after school drop-off or combining them with existing daily routines further reduced barriers to participation and created opportunities for spontaneous encounters with other residents.

*“We feel welcome when it feels warm and cozy.” (W8, municipality 3)*

All women from all municipalities mentioned that learning the Dutch language was an important subject for them and found this a pleasant activity to connect with each other. Neighbourhood workers also mentioned that by understanding the language, women feel more empowered and socially integrated. Possibly, it could contribute to connecting with residents with different cultural backgrounds of the neighbourhood. Some women described that language remains a significant barrier to attend activities. When communication and programming are offered only in Dutch, participation becomes difficult.

The diversity among women, ranging in backgrounds, needs, and levels of mobility, requires more nuanced outreach. Women described that they sometimes searched for women with a mutual background. This was also expressed by neighbourhood workers. At the same time, some women also expressed that they were interested and open to getting to know women from other different cultures. Despite different cultural backgrounds, it was emphasized by the women that eating and cooking together brings people together. Moreover, informal (walk-in) coffee mornings made it easier to connect with neighbours from diverse backgrounds.

Women expressed that those who tend to stay at home or experience loneliness are harder to reach and especially unlikely to enter a community centre spontaneously. Despite the offerings of available activities, personal and culturally sensitive communication is essential to reach them. Simply receiving a flyer in the mailbox is often not sufficient to encourage participation. Therefore, some women asked neighbours to accompany them to certain activities to engage more easily. This was suggested by the local community centres as well. A neighbourhood party was also suggested in multiple municipalities as an easy and accessible activity for people to connect.

*“In that apartment building, there is a lot of loneliness. And for many people and residents, it’s difficult to take the step to come here [community centre]. Because they feel ashamed, they feel like they are being watched, and they don’t feel at ease.” (W3, municipality 3)*

Lastly, a key element in the offering of activities is that there is a local initiator or a neighbourhood worker who is able to reach the women and understands the needs and wishes of the women. In this case, the activities are more tailored to this group. In addition to this, a familiar face creates trusts and improves the feeling of safety. Familiar faces and perceiving the space as ‘theirs’ - a place where they could contribute – were key factors for feeling comfortable and motivated to interact with others. On the other hand, participants explained that having one key initiator to rely on makes it vulnerable in case this person withdraws. It carries the risk of losing the engagement of residents.

Overall, women described successful activities as those that combined their interests, practical accessibility, meaningful social interaction, personal outreach, a local initiator, and cultural sensitivity. Together, these elements contributed to feelings of trust, belonging, and confidence to participate.

#### Activities for teenage girls.

Teenage girls expressed a desire for more age- and gender-specific activities. When such activities were offered, they were well-received. Girls preferred informal, group-oriented formats such as chatting, cooking, baking, watching movies and playing sports such as football, preferably in girls-only settings. Activities such as henna nights or makeup sessions were also welcomed. Public spaces like malls or playgrounds served as informal hangouts. Girls appreciated being able to socialize without financial expectations. A pleasant, welcoming atmosphere was essential.

*“What I notice is that let’s say, the little children in my neighbourhood have activities at the community centre quite often. It’s super fun. And for example, they do things like painting nails or crafting. I don’t know. Well, maybe they should do something a bit more for our age too. I don’t know if it exists yet, but maybe then posters could be put up so that you're a bit more informed.” (TG 9, municipality 2)*

#### Communication.

Both women and girls reported that unclear communication hindered participation and therewith social interaction. Many were unaware of available activities. Neighbourhood workers expressed this similarly; and added the example that it was possible for residents to rent a space for free, however many did not know about it.

Women relied heavily on neighbourhood workers and word-of-mouth for information. Flyers, WhatsApp groups, and community apps also helped, especially when information is offered in multiple languages. Teenage girls preferred digital communication, such as Instagram or WhatsApp, often facilitated by youth workers. In addition, mothers could serve as information channels for girls. Personal invitations, local platforms, and welcoming newcomers with relevant information were suggested ways to improve outreach.

Next to information regarding activities, women indicated that they want to be informed about the available services in the neighbourhood and know where and who to turn to with their questions. The women also expressed a desire for accessible information on parenting, health, and finances. According to the participants, multifunctional venues like libraries allow anonymous access to sensitive services such as finances. In addition, the presence of multiple organizations in one building was seen as helpful for quick support by neighbourhood workers. However, unclear communication, especially due to language barriers, was a common challenge in providing accessible information.

*“That information is so important. It is important that you know where to go. What is it? Where can you turn to if something happens? Some women are learning Dutch, and then that information is often hard to find.” (W4, municipality 1)*

#### Unorganized and spontaneous meetings.

Next to organized social activities in the neighbourhood, informal, spontaneous meetings and social interactions were of great importance to bring a feeling of home and safety to the women. Participants described the neighbourhood itself as a social environment in which accessible public spaces and everyday routines facilitated spontaneous encounters that gradually strengthened social relationships, trust, and feelings of belonging. Doing groceries, seeing each other at for example the marketplace created recognition and having such a sense of belonging in the neighbourhood. The women appreciated getting to know each other and normalizing greeting each other and neighbours on the street. As a consequence, asking or providing help to each other was then easier and highly appreciated.

*“I always greet my neighbours. Always greeting, and they also come to bring food.” (W5 municipality 3)*

#### Social control and supervision.

In public spaces, women described the presence of supervision and social oversight, for instance by community figures such as ‘neighbourhood mothers and -fathers’, as positively contributing to the feeling of safety, since it helps prevent crime and loitering youth. This encouraged participants to use public spaces more. These parents are volunteers from the local neighbourhood who monitor activities, interact with youth, and establish connections between residents and institutions. This was particularly important in local play areas, which was confirmed by neighbourhood workers. Neighbourhood workers and women expressed that these familiar faces in the community also help build trust among residents. For teenage girls, the presence of youth workers who can keep an eye on the situation was seen as reassuring and supportive.

*“During New Year's, we always have neighbourhood fathers and that helps a lot to keep things calmer in the neighbourhood.” (TG 8, municipality 2)*

#### Desire to engage and contribute to improve the neighbourhood.

Many women expressed a strong willingness to contribute to the neighbourhood, especially in support of other women, new residents, or children. Teenage girls also wanted to be involved, to be heard and acknowledged, and their ideas to be utilized. Some neighbourhood workers were open to work together with teenage girls, for instance to create a group of teenage girls to work together to improve the neighbourhood.

*"We, girls, think it is important that professionals take our ideas seriously. And that we receive feedback when we have given input." (TG6, municipality 4)*

*"We feel very involved in the neighbourhood and are truly willing to contribute." (W13, municipality 1)*

The willingness of the women and teenage girls to engage in the neighbourhood was strongly influenced by time, money, family life, and daily tasks. A sense of autonomy and responsibility for a place, activity, or task was described as creating ownership.

#### Key role for neighbourhood workers to listen, act, facilitate and empower.

As mentioned above, women and girls expressed the need to be seen and heard in their ideas and the desire to contribute and improve the neighbourhood. Therefore, it was important for them to know where to go to share their ideas. Helpful communication is a fixed, and trusted point of contact in the neighbourhood. Neighbourhood workers who are close to these groups are in a unique position to listen carefully and respond to their wishes, needs, and talents and eventually connect with other organisations. For many women, it is particularly easy if it is a woman with a similar background and easy to reach, which fosters recognition and mutual understanding. Neighbourhood workers mentioned that youth councils or designated moments for youngsters to speak up are valuable opportunities to gather input from this group.

*“We, girls, can suggest it to [youth worker]. (..) And yes, we are involved and especially encouraged to do that. From [youth worker], it’s like, “Hey, if you have a good idea, it’s accepted, and then we can organize it.” So that’s really great. And so, it happens.” (TG7, municipality 4)*

Neighbourhood workers also acknowledged the importance of putting women and teenage girls at the centre of approach and empower them by encouraging them to use their talents for the benefit of the community. For instance, giving women a role as ambassadors or co-organizers. This creates a shared ownership and responsibility between the women or teenage girls themselves and the involved organisations, such as welfare institutions and social workers. Women indicated that support from organisations and neighbourhood workers is critical and should come in the form of materials, funding, time and space. Women then were also more informed how to organize something and for instance, asking for budget or reserving a space in the local community centre.

Together, the data showed that neighbourhood workers fulfil multiple roles simultaneously: they facilitate communication, strengthen participation, connect organisations and residents, and help create the trust and confidence necessary for residents to become actively involved in their neighbourhood.

#### Transparency and feedback.

Another aspect the women and teenage girls both mentioned is the importance of receiving feedback from the municipality from their input.

*“Well, actually, it’s nice if it’s shown that something is being done with our information, because we're often asked, “What would you like?” But yeah, it would be nice if they at least let us know they've heard it or something.” (TG3, municipality 4)*

Transparency regarding bureaucratic procedures can increase understanding about why municipal processes may move slowly and why the impact may not be immediately visible at the local level, according to the participants. For instance, when concerns are raised with the municipality or housing corporation, they were sometimes perceived as not being followed up. It was unclear to women whether there was no action taken, or whether action was not feasible. Next to this, women appreciated being acknowledged in a meaningful way when they contributed to organising neighbourhood activities. Recognition should be in balance with their level of involvement and tailored to the individual’s contribution and commitment to the community.

#### Collaboration.

Finally, strong long-term collaboration was emphasized by the neighbourhood workers. They described that when organisations actively connect and collaborate with each other, they can create a safety net for residents’ questions, ideas, concerns, or possible incidents. The social network in the neighbourhood becomes vulnerable if the engagement in the neighbourhood is strongly dependent on one or two initiators. For instance, neighbourhood workers expressed the possibility to strengthen collaboration between housing corporations, neighbourhood workers and welfare organisations. For example, housing corporations have sight on the new residents and neighbourhood workers could anticipate on organizing activities for this new neighbour. When issues such as financial difficulties or housing-related problems are present, enhanced collaboration between organisations can improve the provision of appropriate support. The exchange of knowledge, materials, and time among organisations was desired, along with a shared vision for both groups.

#### Creative use of available spaces.

Women and neighbourhood workers highlighted the potential of creatively repurposing vacant or underused spaces for community use. Collaboration between organizations and municipalities was seen as key to realizing this potential.

#### Overview of the themes mapped onto the domains.

Table 2 provides an overview of how the themes identified in this study relate to the four domains: hardware, software, orgware, and mindware. The table illustrates how each theme encompasses one or more of these domains. Empty cells indicate that no relevant findings were identified for that specific domain within the corresponding theme, rather than the absence of that domain in the framework. The overview is intended to support the interpretation of the findings by illustrating how the themes relate to the four interconnected domains.

**Table 2:** Overview table of the mapping of the themes onto the four domains.

Theme	Hardware	Software	Orgware	Mindware
**Attractive and accessible spaces**	Clean, accessible, spacious, suitable and well-maintained indoor and outdoor spaces; green areas; kitchens; visibility of children; privacy	Enabling social interaction and activities to take place	Maintenance and management of spaces	Feeling welcome, comfortable, safe, a sense of belonging, user-friendliness and ambiance
**Amenities located close to home**	Nearby parks, playgrounds and community centres	Daily encounters during neighbourhood routines	—	Accessibility and ease of participation, and lively atmosphere
**Sufficient and strategically located seating places**	Benches, picnic tables, shaded seating, sheltered seating	Informal conversations, supervision of children and eating together	Placement of seating and maintenance	Comfort, privacy and perceived low threshold
**Safe and diverse play and sports areas**	Age-appropriate playgrounds, sports facilities, outdoor fitness equipment, drinking water taps	Playing and exercising together	Maintenance	Feeling safe, welcome and accepted
**Activities for women**	Child-friendly meeting places and venues for activities	Cooking, walking, sports, language classes, coffee mornings, cultural activities and informal conversations	Neighbourhood workers tailoring activities, personal outreach and familiar faces	Trust, belonging, cultural recognition, confidence to participate and feeling welcome
**Activities for teenage girls**	Girls-only spaces	Group activities, sports, creative activities and informal gatherings	Youth workers facilitating activities	Feeling comfortable, represented and socially connected
**Communication**	Accessible information points and multifunctional venues	Communication through WhatsApp, Instagram, flyers and community networks	Neighbourhood workers, youth workers and local organisations	Knowing where to go, trust and reduced participation barriers
**Unorganized and spontaneous meetings**	Public spaces, shops and marketplaces	Everyday encounters and greeting neighbours	—	Sense of home, familiarity and belonging
**Social control and supervision**	—	—	Neighbourhood mothers/fathers and youth workers	Safety, trust, support and reassurance
**Desire to engage and contribute to improve the neighbourhood**	—	—	Resident participation and collaboration with professionals, co-organising activities	Ownership, responsibility and feeling heard
**Key role for neighbourhood workers to listen, act, facilitate and empower**	—	Supporting resident initiatives and co-organizing activities	Listening to, connecting with, and facilitating and empowering residents	Trust, recognition, ownership, responsibility and self-efficacy
**Transparency and feedback**	—	Communication with residents	Co-organizing activities, recognition of residents, and giving feedback	Feeling acknowledged and valued
**Collaboration**	—	—	Collaboration between organisations and joint neighbourhood initiatives	Continuity and confidence in local support
**Creative use of available spaces**	Repurposing vacant or underused spaces	New opportunities for meeting	Collaboration between municipalities and organisations	—

## Discussion

This study aimed to explore which elements make neighbourhood meeting places work for women with a non-Western migration background and teenage girls in disadvantaged neighbourhoods. The findings of this study support recent placemaking perspectives that conceptualise neighbourhood meeting places as the outcome of interactions between physical characteristics, social practices, organisational arrangements, and residents’ perceptions and experiences [29,30,32]. Physical environments only became meaningful when supported by appropriate activities, organisational facilitation, and participants’ experiences of, among others, safety, belonging and ownership. Applying the BVO model beyond its original focus on physical activity demonstrated that neighbourhood meeting places for women with a non-Western migration background and teenage girls are shaped by an interplay between hardware, software, orgware, and mindware. In particular, the findings suggest that perceptions of feeling welcome, safety, trust and belonging are not merely outcomes of successful meeting places but serve an essential dimension in their functioning.

The findings on mindware also align closely with established frameworks in environmental and community psychology. The concept of place attachment, defined as the bond people form with meaningful environments through affective, behavioural, and cognitive ties, highlights how such emotional connections foster identity, belonging, and long-term commitment to a place [35,36]. Scannell and Gifford [37] conceptualise place attachment as an interaction between the person, psychological processes, and place characteristics, illustrating how emotional and cognitive experiences of places emerge from both their physical and social environments. Manzo and Perkins [35] further argue that strong place attachment can motivate collective action to improve one’s community. In parallel, psychological ownership – the feeling that a space is ‘ours’ - has been shown to significantly predict individuals’ intentions to visit and maintain public spaces and goods [38]. Together, these findings suggest that mindware represents an important conceptual addition to the BVO framework. While hardware, software and orgware describe the physical, social and organisational conditions of meeting places, mindware captures how these conditions are perceived by residents. Incorporating mindware therefore enables a more comprehensive understanding of why some neighbourhood meeting places become meaningful, inclusive and frequently used, while others remain underutilised despite comparable physical or organisational characteristics.

Further, this study emphasizes the resilience and connectedness within so-called disadvantaged neighbourhoods. Despite the negative image of these neighbourhoods and the challenging conditions that these neighbourhoods can bring for its residents, this study reveals that many women and girls hold a positive drive to contribute to local improvements. A result that is also found in other studies on community participation among immigrants [45–47]. Notably, groups often seen as less visible, such as women with a non-Western migration background and teenage girls, do show a willingness to take part in social interaction and community activities. This suggests that their limited visibility should not always be mistaken for a lack of interest and emphasizes the need to engage these groups meaningfully.

Across all dimensions (the four wares), women and teenage girls shared several values regarding meeting spaces. Firstly, both emphasized the importance of clean, well-maintained, safe, and green public environments. Also, safety concerns, due to traffic, crime, or loitering youths, were mentioned by both women and girls, often leading to avoidance of outdoor spaces, especially in the evenings. These findings align with research that highlights how women’s and girls’ sense of safety in public spaces is shaped by physical conditions such as lighting, visibility, and greenery, which influence their confidence in using these spaces [48–53]. Secondly, both groups appreciated accessible seating, particularly benches near playgrounds (women) or more private, sheltered seating (girls). Previous studies have also highlighted the need for appropriate seating areas [25,52,54,55]. For example, research has found that seating for girls should be designed in a manner that allows them to face one another [25], and that seating space should not be situated in such a way that it could trap them or provide hiding places for potential attackers [52]. Moreover, Subramanian et al. [55] writes that, by designing seating areas in accessible and inviting ways for girls, the girls are not perceived as troublesome, but rather as active and legitimate participants in public space, addressing a concern expressed by several girls in this study. Lastly, proximity to essential daily amenities, such as schools and shops, was also valued by both groups, supporting theories of place-making that highlight the importance of integrating meeting places into existing neighbourhood routines to enhance social interaction and local vibrancy [56].

For women, child-friendly design and visibility for supervision are of importance. The study of Li et al. [54] also highlighted the importance of adding supportive facilities tailored to women’s needs in public spaces. For instance, to accommodate mothers with young children, designated areas could provide private and convenient lactation and diaper-changing rooms, as well as spaces to entertain children during activities [54]. In terms of programming, women in the current study particularly appreciated activities such as cooking and eating together, sewing, learning the Dutch language, and informal gatherings like coffee mornings. They emphasized that providing childcare or aligning activities with school hours would improve participation. These findings align with literature highlighting the importance of culturally relevant, collective food-based programming, particularly when supported by practical measures such as childcare [57]. Furthermore, Mills et al. [58] explains that women’s participation in cooking-related community activities is strongly shaped by their available time and daily routines, reinforcing the need to adapt activities to school schedules and childcare needs.

The results of the current study regarding the needs of teenage girls align with literature showing that public spaces are frequently designed in ways that favour boys, leaving girls feeling excluded or unsafe [23,25,53,55]. Gender-inclusive urban design, such as designated girl-only areas and ‘chill’ zones, has been found to improve girls’ engagement, autonomy, and sense of belonging. The studies of Li et al. [54], Subramanian et al. [55], and Hjort and Larsen [59] reflect the need of the teenage girls for autonomy, peer bonding, informal use of space without economic barriers and girl-only zones. At the same time, other studies suggest that social cohesion can also be strengthened through inclusive initiatives that foster shared experiences, for instance via intergenerational or gender-diverse programming [60–62]. Future research could further explore how both girl-specific and cross-group interventions contribute to more inclusive neighbourhood environments.

A lack of clear, visible information and thus lack of awareness regarding local initiatives is common in (disadvantaged) neighbourhoods, according to previous research [63,64]. For instance, the study of Swart et al. [63] found that residents in low-income neighbourhoods often report a lack of awareness regarding local opportunities, as formal communication rarely reaches them, and existing channels are not trusted or accessible. Community participation via religious organizational membership was found common, indicating that communication via religious organizations has potential for reaching residents – a communication tool that was not mentioned in the current study. According to the current study, women preferred personal contact via neighbourhood workers and word of mouth. The preference for personal contact is supported by Froonjian and Garnett [65], who emphasize that reaching ‘hard-to-reach’ populations is most effective through trusted intermediaries and informal communication channels, such as family, peers, or ethnic media, rather than top-down messaging. Similarly, Rishbeth [66] argues that public spaces and outreach strategies must be culturally inclusive and recognizable for ethnic minority groups, and that leveraging symbolic familiarity through trusted relationships can enhance feelings of welcome and accessibility.

Teenage girls, however, preferred digital tools like Instagram and WhatsApp. The use of Instagram as communication tool to reach adolescent girls has been researched before by Thomas et al. [67], who conducted a pilot study demonstrating that a youth-focused Instagram account could enhance adolescent participation in health programs. After three months, 43% of the interviewed girls reported that the account motivated them to continue attending [67]. The study of Dai and Liu [68] not only confirms digital tools as suitable communication channels but also suggests that digital environments may also serve as extensions of physical meeting spaces or even as independent social arenas that support interaction and belonging. This digital dimension was not thoroughly explored in the current study and could serve as input for future research.

Existing literature confirms the importance of citizen participation. People feel more connected to meeting places when they have a say in how these spaces are shaped and programmed [56,69,70]. Actively involving residents thus leads not only to more tailored interventions but also fosters stronger connections to the neighbourhood and more sustainable engagement with meeting places. Furthermore, it is essential to place women and teenage girls at the centre and empower them by encouraging them to use their talents for the benefit of the community [71,72]. Assigning them roles as ambassadors or co-organizers enables them to make meaningful contributions. Therefore, effective meeting places require both a targeted and participatory approach. In addition to citizen participation and empowerment, the current study also found that transparency and feedback from municipalities and neighbourhood workers is important to foster trust and understanding among women and teenage girls. Research also shows that transparency increases trust in government institutions [73–76]. Additionally, citizen participation enhances this effect by improving perceptions of transparency and responsiveness [74]. These studies thus confirm the importance of transparency and feedback in citizen participation and engagement.

The findings of this study also contribute to a broader shift in the literature on public space and neighbourhood design. Although research on public spaces and meeting places has expanded considerably over recent decades, urban planning and design have historically been based on the experiences and mobility patterns of a presumed ‘average’ user, which in practice has often reflected the needs of adult men rather than those of women or other underrepresented groups [77,78]. As a result, aspects that are particularly important to women, such as perceived safety, caregiving responsibilities, accessibility during daily routines, and opportunities for informal social interaction, have often received less attention in both research and urban planning [77,79]. More recently, scholars have argued that creating inclusive cities requires recognising that different groups experience and use public space in fundamentally different ways, and that planning should therefore move beyond one-size-fits-all approaches [80,81].

Furthermore, while the majority of existing literature on meeting places and social cohesion used in this discussion focuses on women in general, it offers limited insights specific to women with a non-Western migration background. However, the findings from the current study suggest that the differences between women with a non-Western migration background and other women, such as Western women, may not be as pronounced as might expected. Their needs might majorly align with women in general. Across both groups, similar values emerged: the importance of safety, proximity, cleanliness, child-friendly environments, and opportunities for informal interaction. This possible alignment may indicate that many of the core conditions for meaningful meeting places transcend cultural or ethnic background. Nevertheless, the findings also demonstrate that inclusivity requires attention to the ways in which these shared needs are addressed. Cultural inclusivity – including language, culturally appropriate activities, trusted communication channels, and recognising different perceptions of public space – remains essential to ensure that neighbourhood meeting places are experienced as welcoming and accessible by diverse groups.

### Strengths and limitations

This study has several strengths. It employed a combination of qualitative methods, including interviews, PhotoVoice, and focus groups, which provided a rich and nuanced understanding of the experiences and needs of women with a non-Western migration background and teenage girls, as well as the perspectives of neighbourhood workers. However, using these methods may also have introduced different forms of response bias. Focus groups stimulated collective reflection and enabled participants to build on each other’s experiences, but group dynamics and perceived social norms may have discouraged the expression of dissenting or sensitive views. Conversely, individual and paired interviews may have provided greater opportunities for discussing personal experiences, yet participant–researcher power dynamics could have affected participants’ willingness to speak openly, especially in interviews involving professionals or interpreters. The use of multiple methods therefore represents both a strength, by enabling participation of diverse groups under varying circumstances, and a limitation, as each method may have generated different blind spots in the data.

Strengths can also be found within the target groups, as a diverse mix of ages, backgrounds (in case of the women), and experiences was represented, enhancing the validity of the findings. Finally, conversations around neighbourhood meeting places initiated during the data collection phase led to new connections among participants and stimulated local dialogue, contributing to emerging social cohesion.

Nonetheless, several limitations should be noted. First, there was selective participation: among the women, participants were primarily mothers, which may limit the applicability of the findings to women without children. Furthermore, representation across the participating municipalities was uneven due to the recruitment process being more or less challenging in the different neighbourhoods, restricting comparability between contexts. Also, initially the study was communicated as involving a four-hour session, which proved too long for many participants. The format was therefore shortened to two hours to improve accessibility. This initial duration may have discouraged some potential participants from signing up. In addition, in some cases, language barriers also limited the depth of conversation and expression. These limitations were mitigated to some extent through validation sessions in which the results were discussed with participants prior to final analysis.

Lastly, PhotoVoice was conducted only with the group of women. While the method was initially intended for both groups, implementation among teenage girls proved unfeasible due to limited interest, time constraints, difficulties in reaching girls, and the requirement for signed parental consent for participants aged 12–16 that formed an extra barrier for the girls. This suggests a need for alternative approaches in future research. Consequently, data saturation may not have been reached for this group, and the depth of analysis for teenage girls remained more limited.

## Conclusion

This study demonstrates that a meaningful meeting place where women with a non-Western migration background and teenage girls feel comfortable and are motivated to spend time, emerges when elements of hardware, software, orgware, and mindware are aligned and mutually reinforcing. Co-creation and the empowerment of women and teenage girls is of importance to achieve this. Creating such meeting places requires the design of accessible and welcoming spaces, offering activities that are culturally and age appropriate, clear and inclusive communication, overcoming language barriers, and feelings of belonging and safety. Equally important is establishing a robust network of residents, neighbourhood workers, and local authorities with clearly defined roles, and fostering a culture of trust, inclusion, and ownership. In doing so, neighbourhoods can evolve into places where everyone feels welcome, is encouraged to contribute their talents, and where sustainable social networks can flourish.

## Supporting information

S1 Table Coding tree. (DOCX)
