# Supplementary material for: Looking through different lenses at meeting places in disadvantaged Dutch neighbourhoods: A qualitative participatory study assessing needs, barriers and success elements for women with non-Western migration backgrounds and teenage girls
Source: PLoS One. 2026 Sep 9;21(9):e0356072. doi: 10.1371/journal.pone.0356072 (PMC13557403; doi:10.1371/journal.pone.0356072)
Supplement: Supplementary file 1 [file pone.0356072.s001.docx]

| **Code System (groups, subgroups, codes)** | | | | **Memo** |
| --- | --- | --- | --- | --- |
| Context |  |  |  |  |
| Ideas for processing of results |  |  |  |  |
| Chatting, not relevant |  |  |  | These are passages where the participants and/or researchers were chatting, in between research activities. No coding is required. |
| Positive |  |  |  |  |
| Negative |  |  |  |  |
| Mindware |  |  |  | Definition: behaviour, values, perception, and emotions of people. |
|  | Art |  |  |  |
|  | Shopping |  |  |  |
|  | Meaning-making |  |  |  |
|  | Sports and physical activity |  |  |  |
|  | Cooking |  |  |  |
|  | Children |  |  |  |
|  | Sense of belonging |  |  |  |
|  | Time |  |  |  |
|  | Learning |  |  |  |
|  | Relaxation |  |  |  |
|  | Connection |  |  | Also friendships. |
|  | Helping |  |  |  |
|  | Health |  |  |  |
| Hardware |  |  |  | Definition: physical location, physical (outdoor) space. |
|  | Privacy |  |  | It can be indoors, in its own separate room, but it can also be outdoors in a sheltered spot. |
|  | Outdoor spaces |  |  |  |
|  |  | Physical places |  |  |
|  |  |  | Park |  |
|  |  |  | Swimming pool |  |
|  |  |  | Square |  |
|  |  |  | Shopping centre |  |
|  |  |  | Picnic table |  |
|  |  |  | Church |  |
|  |  |  | Community centre |  |
|  |  |  | School |  |
|  |  |  | Playground |  |
|  |  | Art |  |  |
|  |  | Multifunctional spaces |  |  |
|  |  | Accessibility |  |  |
|  |  |  | Central |  |
|  |  |  | Nearby |  |
|  |  | Design |  |  |
|  |  | Level of crowding |  |  |
|  |  | Availability of physical space |  |  |
|  |  | Clean |  |  |
|  |  | Well-maintained |  |  |
|  |  | Seating areas |  |  |
|  |  | Greenery |  |  |
|  |  | Shelter |  |  |
|  |  | Public safety |  | Definition: the extent to which someone feels safe in public space (in their physical environment). |
|  | Indoor spaces |  |  |  |
|  |  | Multifunctional spaces |  |  |
|  |  | Accessibility |  |  |
|  |  |  | Central |  |
|  |  |  | Nearby |  |
|  |  | Design |  |  |
|  |  | Level of crowding |  |  |
|  |  | Availability of physical space |  |  |
|  |  | Clean |  |  |
|  |  | Well-maintained |  |  |
|  |  | Seating areas |  |  |
|  |  | Windows |  |  |
| Software |  |  |  | Definition: Activities offered, information and communication, guidance, interaction. |
|  | Social control |  |  |  |
|  | Neighbourhood image |  |  |  |
|  | Norms and values |  |  |  |
|  |  | Street culture |  |  |
|  | Information provision |  |  |  |
|  | Cultural background |  |  |  |
|  | Social safety |  |  | Definition: Social safety is a broad and complex concept that focuses on the extent to which individuals feel safe in social interactions and in their social environment. |
|  | Proficiency in the Dutch language |  |  |  |
|  | Recognizability |  |  |  |
|  | Trust |  |  |  |
|  |  | Self-confidence |  |  |
|  |  | Trust in others |  |  |
|  | Continuity |  |  |  |
|  | Activities |  |  |  |
|  |  | Art |  |  |
|  |  | Shopping |  |  |
|  |  | Meaning-making |  |  |
|  |  | Sports and physical activity |  |  |
|  |  | Cooking |  |  |
|  |  | Children |  |  |
|  |  | Sense of belonging |  |  |
|  |  | Time |  |  |
|  |  | Learning |  |  |
|  |  | Relaxation |  |  |
|  |  | Connection |  |  |
|  |  | Helping |  |  |
|  |  | Duration |  |  |
|  |  | Health |  |  |
|  |  | Childcare possibilities |  |  |
|  |  | Guidance |  |  |
|  |  | Alignment with target group |  |  |
|  |  | Combining activities |  |  |
|  | Atmosphere |  |  |  |
|  | Obligation |  |  |  |
|  | Accessibility (low-threshold) |  |  |  |
|  |  | Costs |  |  |
|  |  | Drop-in possibilities |  |  |
|  | Hospitality |  |  |  |
|  | Communication |  |  |  |
| Orgware |  |  |  | Definition: Policy and vision, involved organizations, ownership and finances, monitoring and evaluation, maintenance and management, collaboration and citizen participation |
|  | Entrepreneurship |  |  |  |
|  | Willingness |  |  |  |
|  | Appreciation |  |  |  |
|  | Understanding |  |  |  |
|  | Coordination |  |  |  |
|  | Support |  |  |  |
|  |  | Funding |  |  |
|  |  | Resources |  |  |
|  |  | Support |  |  |
|  | Bureaucracy |  |  |  |
|  | Bottom-up |  |  |  |
|  | Top-down |  |  |  |
|  | Collaboration |  |  |  |
|  | Involved residents |  |  |  |
|  | Involved organizations |  |  |  |
|  |  | Police |  |  |
|  |  | Municipality |  |  |
|  |  | Welfare organization |  |  |
|  |  | Housing corporation |  |  |
|  | Ownership |  |  |  |
|  | Responsibility |  |  |  |
